# Supplementary material for: Insignificant Difference in Biocompatibility of Regenerated Silk Fibroin Prepared with Ternary Reagent Compared with Regenerated Silk Fibroin Prepared with Lithium Bromide
Source: Polymers (Basel). 2022 Sep 18;14(18):3903. doi: 10.3390/polym14183903 (PMC9502819; doi:10.3390/polym14183903)
Supplement: Supplementary file 1 [file polymers-14-03903-s001.zip › polymers-1890173-supplementary.pdf]

Supplementary Materials

Insignificant Difference in Biocompatibility of Regenerated Silk Fibroin Prepared with Ternary Reagent Compared with Regenerated Silk Fibroin Prepared with Lithium Bromide

Guotao Cheng [1], Xin Wang [2], Mengqiu Wu [1], Siyuan Wu [1], Lan Cheng [1], Xiaoning Zhang [1] and Fangyin Dai [1,*]

[1] State Key Laboratory of Silkworm Genome Biology, Key Laboratory of Sericultural Biology and Genetic Breeding, Ministry of Agriculture and Rural Affairs, College of Sericulture & Textile and Biomass Sciences, Southwest University, Chongqing 400715, China
[2] Center for Joint Surgery, Southwest Hospital, Third Military Medical University (Army Medical University), Chongqing 400038, China
* Correspondence: fydai@swu.edu.cn

Quantitative Analysis of Secondary Structure

Select the amide 1 region (1700cm^{-1}-1600cm^{-1}) of the FTIR spectrum for quantitative analysis. After adjusting the baseline, a second derivative calculation was performed on the spectrum to determine the number of peaks. The spectrum can be smoothed to reduce interference. Then use Peakfit software to perform multi-peak fitting, and multiple fittings reduce residuals. The secondary structure content of silk fibroin was calculated based on the area of the fitted peaks. Peak assignments refer to the Table S1. The fitting results are shown in Figure S1.

Table S1 Vibrational band assignments for the amide I region of silk fibroin [53]

Wavenumber range (cm^{-1})	Secondary structure assignment
1616-1627	β-sheets
1628-1637	β-sheets
1638-1646	Random coils
1647-1655	Random coils
1656-1662	α-Helix
1663-1670	Turns
1671-1685	Turns
1686-1695	Turns

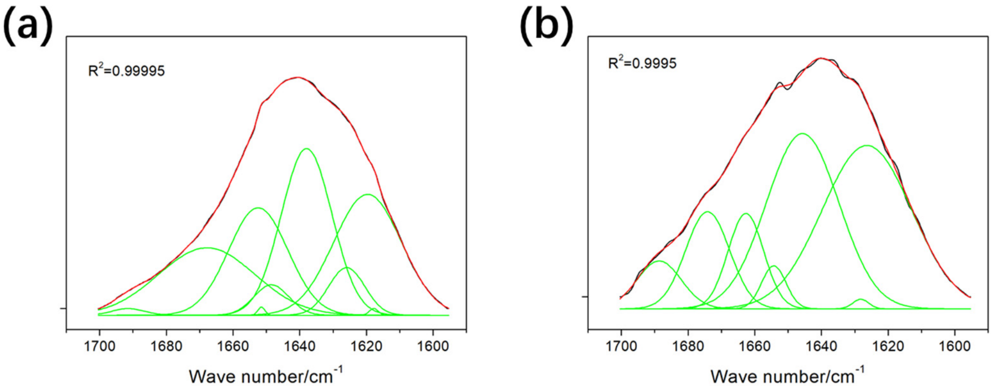

Figure S1: Multimodal fitted curves of the FTIR spectra of RSF-Ca (a) and RSF-Li (b). The black curve is the original spectrum, the red is the fitted total spectrum, and the green is the individual peaks. R^2 is the residual value.

Roughness of the RSF film

The roughness of the RSF film was observed and analyzed by AFM. The result is shown in Figure S2.

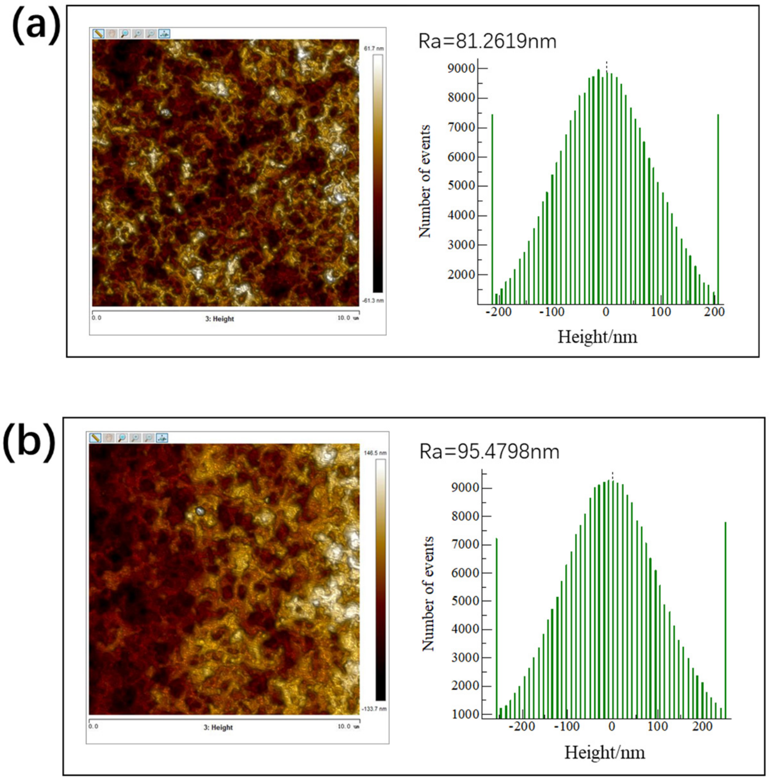

Figure S2: The AFM photos and roughness analysis of RSF-Ca (a) and RSF-Li (b).

Platelet morphology

To ensure that the adhered particles were rabbit platelets, we performed higher magnification observations. It was observed that the particles adhered to the RSF film were oblate or irregular star-shaped, with a size of 2 μm, which was a typical rabbit platelet morphology (Figure S3).

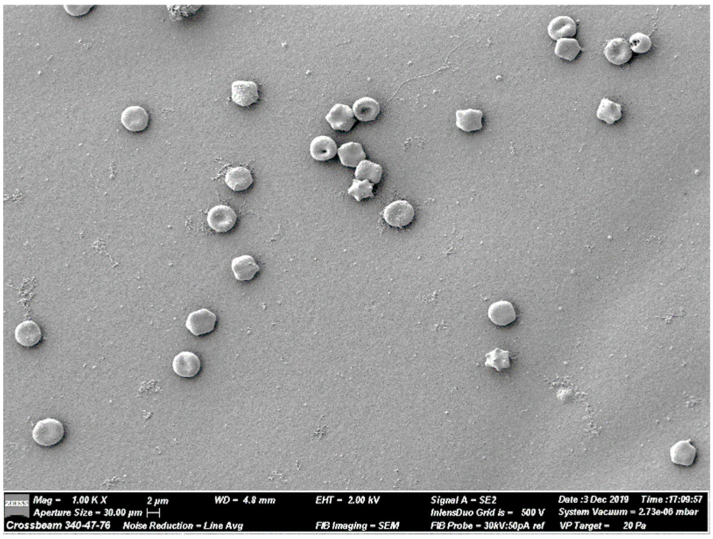

Figure S3: Morphology of rabbit platelets adhered to the RSF film.

References

[53] Brian D. Lawrence, Fiorenzo Omenetto, Katherine Chui, David L. Kaplan.

Processing methods to control silk fibroin film biomaterial features [J]. Journal of Materials Science, 2008, 43(21) :6967.
